# Successful management of acute thromboembolic disease complicated with heparin induced thrombocytopenia type II (HIT II): a case series

**DOI:** 10.1186/1477-9560-6-9

**Published:** 2008-07-02

**Authors:** Eva Serasli, Maria Antoniadou, Venetia Tsara, Vassilis Kalpakidis, Angelos Megalopoulos, George Trellopoulos, Georgia Papaioannou, Pandora Christaki

**Affiliations:** 1 2nd Department of Chest Medicine, General Hospital G. Papanikolaou, Thessaloniki, Greece; 2 Department of Interventional Radiology, General Hospital G. Papanikolaou, Thessaloniki, Greece; 3 Cardiovascular Department, General Hospital G. Papanikolaou, Thessaloniki, Greece; 4 Hematology Department, Papageorgiou General Hospital, Thessaloniki, Greece

## Abstract

Heparin-induced thrombocytopenia type II (HIT II) is a rare immune-mediated complication of heparin. The diagnosis of HIT is considered in patients exposed to heparin, presenting with thrombocytopenia and thrombosis.

We present two cases with massive pulmonary embolism and HIT, successfully treated with the administration of fondaparinux, an alternative anticoagulant, combined with the insertion of an inferior vena cava filter for the prevention of new thromboembolic events. The two cases supplement the available data of the use of fondaparinux in patients with HIT and pulmonary embolism, before further large studies establish its efficacy and safety in this group of patients. Moreover, the management of these patients reveals the need for future evaluation of the combined therapy of alternative anticoagulant agents with the placement of vena cava filters.

## Background

Heparin in its two forms (unfractionated heparin and low-molecular weight heparin) remains the mainstay of treatment for venous and arterial thromboembolism. Heparin-induced thrombocytopenia type II (HIT II) is a well documented, immune mediated severe adverse effect of heparin, caused by IgG (more rarely IgM) antibodies against the heparin/platelet factor 4 (PF4) complex, resulting in platelet activation and aggregation, endothelian activation, thrombin generation and eventually in thrombocytopenia and thrombotic events [1,2]. For patients with HIT II, the discontinuation of heparin alone is not sufficient and the diagnosis necessitates the administration of an alternative anticoagulant [3-5].

We report the cases of two patients with HIT II and massive pulmonary embolism, successfully managed with fondaparinux sodium, a novel selective inhibitor of activated factor X and the insertion of an inferior vena cava filter.

## Cases presentation

The first case involved a 60-year-old woman with intracranial hemorrhage, hospitalized in our hospital in a stable neurological state. She had a history of a total arthroplasty of her right ischium 25 days ago and mild thrombocytopenia after the administration of low molecular weight heparin, which had been replaced by aspirin. On day 5, she developed acute respiratory failure with clinical signs of right heart failure, due to massive pulmonary embolism and deep venous thrombosis at the level of right iliac, femoral and popliteal veins. At that time, the platelet count was determined at 50.000/μL, disseminated intravascular coagulation was not present and all routine laboratory tests were within normal limits.

In view of thrombocytopenia after the administration of low molecular weight heparin and the thromboembolic events, HIT was clinically suspected. The diagnosis was confirmed three days later, by the positive enzyme immunoassay for antibodies to heparin-platelet factor 4 (Asserachrom HPIA, Diagnostica Stago). Antibody titers were determined on citrated plasma and results were expressed as percent of the absorbance measured at 450 nm with a positive control (1.900 optical density units), representing the value 100%. Antibody titers were estimated to be 87% (cut off for positive value 23%).

**Figure 1:**
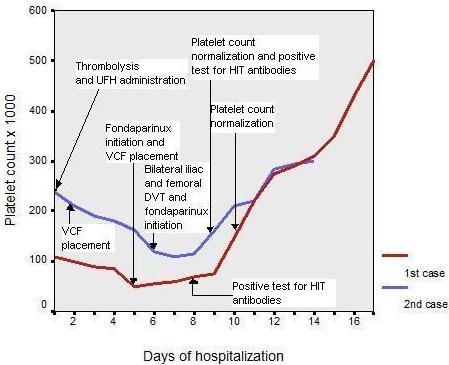
The course of platelet count of both patients during hospitalization in association with clinical events and laboratory confirmation of HIT. Laboratory tests results at the time of the diagnosis, included increased fibrin d-dimer levels (>20 μg/ml and 4.1 μg/ml respectively; normal value <0.5 μg per milliliter) and indicated the absence of disseminated intravascular coagulation (International Normalized Ratio 1.1 and 1.0, respectively; normal range 0.9 to 1.2) without circulating nucleated red cells.

Despite the increased risk of a recurrent intracranial hemorrhage, the presence of severe deep venous thrombosis on the ground of HIT immediated the administration of an alternative anticoagulant and fondaparinux sodium (Arixtra^®^, GlaxoSmithKline) was started subcutaneously in the dose of 7,5 mg daily. Given the severity of the patient's condition and her stabilized neurological status, we decided the administration of the highest recommended dose. Furthermore, the insertion of vena cava filter (Gunther, Tulip™, Cook USA) was decided for the prevention of new embolic events. The clinical status of the patients gradually improved and platelet count normalized 5 days after the initiation of fondaparinux (figure 1). No further bleeding or new thrombosis were observed. The patient was discharged on fondaparinux treatment after 16 days of hospitalization.

The second case was a 32-year-old man, with a medical history of thrombophilia due to hyperhomocystinaemia, hospitalized in our department with massive pulmonary embolism and deep venous thrombosis of his lower extremities, in both popliteal veins. Due to cardiovascular instability and severe respiratory failure, the patient underwent thrombolysis with recombinant tissue plasminogen activator at the dose of 100 mg in 2 hours intravenously (Actilyse^®^, Boehringer) and an inferior vena cava filter (Gunther Tulip™, Cook USA) was inserted. Subsequently, he was given intravenously unfractionated heparin (UFH), accordingly to Rascke protocol (80 U/kg bolus followed by 18 U/kgr/h). At that time the platelet count was determined at 239.000/ml. On day 6, the patient manifested clinical evidence of right lower extremity deep venous thrombosis and in parallel, a decrease in his platelet count to 110.000/μL. The spiral computed tomography of the lower extremities vessels showed new thrombi extending in both iliac and femoral veins up to the level of inferior vena cava filter.

The heparin/PF4 enzyme-linked immunosorbent assay (ELISA) for platelet depended antibodies was strongly positive (65%), confirming the diagnosis of HIT on day 9. Fondaparinux sodium was administrated subcutaneously at the dose of 7,5 mg daily. No further thromboembolic episodes occurred and the platelet count increased steadily three days later (figure 1). The patient was discharged after 14 days of hospitalization, on long-term treatment with fondaparinux.

## Discussion

Heparin induced thrombocytopenia is a rare and serious adverse drug reaction, accompanied with a mortality rate from 18.8 to 50% without treatment [5-7]. In most cases, no other cause for platelet count fall is evident and despite thrombocytopenia, patients manifest new or recurrent venous and/or arterial thrombosis. A retrospective analysis showed that 40–61% of patients with HIT developed thrombosis after the cessation of heparin alone [8]. It is of note that venous thromboembolism (VTE) is infrequently associated with HIT in LMWH treated patients (<1%) [9].

Regarding the therapeutic management of HIT, the currently available alternative anticoagulants are lepirudin, argatroban and danaparoid. Among these agents, lepirudin and argatroban are the treatments approved by the FDA for managing HIT and only lepirudin is available in Greece. However, lepirudin requires monitoring, as the risk of major hemorrhage is directly related to APTT ratio and renal function [10].

Fondaparinux sodium is a new synthetic analogue of the binding site within heparin specific for antithrombin III. Its pharmacokinetic properties allow for a simple once-daily regimen, in a dose between 2.5 and 7.5 mg subcutaneously, without monitoring [7]. Many case reports and retrospective reviews showed encouraging results favouring its use in the therapy of HIT [11]. However, two recent case reports of fondaparinux-related thrompocytopenia and thrombosis [12] and fondaparinux-associated thrombocytopenia in a previous LMWH-HIT [13] respectively highlight the fact that its safety need to be further clarified. A previous study showed that fondaparinux does not activate platelets in the presence of sera obtained from patients with HIT with a statistically significant difference comparing to UFH[14], and recently, Warkentin et al[15] showed that the frequency of formation of anti-PF4/heparin antibodies was the same for orthopedic surgery patients receiving fondaparinux or enoxaparin but the risk of developing HIT was very low.

In hemodynamically stable patients with acute symptomatic pulmonary embolism, once-daily subcutaneously fondaparinux appears to be at least as effective and safe as unfractionated heparin for the initial treatment of pulmonary embolism [16] and in patients with acute symptomatic deep venous thrombosis, fondaparinux was at least as effective and safe as twice-daily body-weight adjusted enoxaparin for their initial treatment [17].

Taking into account the published data and its properties, fondaparinux provided to us a logical alternative anticoagulant for our patients who suffered from HIT, massive pulmonary embolism and deep venous thrombosis.

To our knowledge, there are limited data in the literature for invasive approaches to the treatment of HIT. Emig et al reported the case of a woman with massive thrombosis of the iliofemoral and caval veins in the HIT syndrome [18], who was successfully managed with the combination of transcatheter administration of urokinase and systemic infusion of danaparoid, under the protection of a temporal vena cava filter.

Our first patient developed deep venous thrombosis and massive pulmonary embolism, as a result of HIT syndrome. Given the fact that a recurrent embolic event might have been fatal, the patient required prompt and aggressive management with combined therapy with an alternative anticoagulant and the placement of an inferior vena cava filter. In the second case, the initial treatment of massive pulmonary embolism was thrombolysis, followed by the administration of UFH and the insertion of an inferior vena cava filter. The recurrent deep venous thrombosis up to the level of the IVC filter was the result of the HIT syndrome, which was managed by the administration of fondaparinux. It is thus considered that the cava filter had been proved life saving and prevented new, probably fatal, pulmonary embolism.

There are multiple absolute and relative indications for the use of vena cava filters in VTE [19,20] and concomitant therapy with anticoagulation is typically used in patients with severe cardiopulmonary disease and deep venous thrombosis [20]. Recent data suggest anticoagulation during and after the insertion of vena cava filter for patients who have no contraindications [21]. However, combined therapy with an alternative anticoagulant merits further evaluation, in terms of efficacy, safety, and duration of therapy.

A possible limitation of the study may be considered the expression of the ELISA results, as percent of the absorbance measured at 450 nm with a positive control, instead of using optical density (OD) units. Antibody titers were estimated high, taking into consideration that they were almost four and three fold higher than the cut-off value in both cases. Literature data regarding the association of this method expression with the relative risk of thrombosis are lacking, yet it has recently been applied in order to follow the disappearance of anti-PF4/heparin antibodies under fondaparinux administration in a patient with HIT [22].

## Conclusion

Managing anticoagulation in patients with HIT and massive pulmonary embolism can be extremely difficult and challenging. Fondaparinux may be a viable alternative option, however randomized controlled clinical trials are needed to establish its use in this group of patients, as well as its combination with intravascular insertion of IVC filters for the prevention of embolic events.

## Abbreviations

HIT: Heparin induced thrombocytopenia; IVC: Inferior vena cava filter; VTE: Venous thromboembolism. 

## Competing interests

The authors declare that they have no competing interests.

## Consent

Written informed consent was obtained from the patients for publication of this Case reports and any accompanying images. A copy of the written consents is available for review by the Editor-in-Chief of this journal.

## Authors' contributions

All the authors of the manuscript consisted the medical group who collaborated for the diagnosis and management of the patients. They have all been involved in drafting the manuscript and contributed, each from the point of view of his/her specialty. IVC filters were inserted by VK, AM, GT at the Interventional Radiology Dept of G. Papanikolaou Hospital. All authors have read and approved the final manuscript.
